# Healthy, safe and responsible: the modern female traveller

**DOI:** 10.1186/s40794-021-00141-7

**Published:** 2021-06-05

**Authors:** Irmgard L. Bauer

**Affiliations:** College of Healthcare Sciences, Division of Tropical Health and Medicine, James Cook University, Townsville, QLD 4811 Australia

**Keywords:** Women travellers, Travel health advice, Female hygiene, Menstruation suppression, Tourist behaviour, Risk perception

## Abstract

One-half of all travellers are women; yet, there is a distinct lack of detailed travel health knowledge on topics of unique relevance to women. While there is medical advice relating to stages in the female lifecycle, it neglects women-specific practical aspects despite their ability to harm travellers’ health and cause inconvenience. This paper discusses comprehensively three major aspects of travel as they relate to women. First, it suggests the management of personal hygiene, bodily functions, menstruation and sexual behaviour, and alerts to the limited knowledge on travel mental health issues.

Second, apart from travelling in a female body with its specific demands, being a woman requires special attention to safety and security. Within various travel contexts, women have many opportunities for minimising potential risks.

Finally, guided by travel medicine’s acknowledgment of its role in the concept of responsible travel, this article goes beyond the usual general statements and broad advice and offers detailed and practical suggestions on how the female traveller can contribute to the overall goal of minimising any potential harm to fellow humans and the natural environment. Recognising the scarcity of women-specific travel information, pathways to better education, and a range of suggestions for urgent research facilitate the provision of high-quality travel health care tailored specifically to women’s needs.

*‘There is no reason why a woman cannot go wherever a man goes, and further’*

*(Harriet Chalmers Adams, explorer, 1920)*

## Introduction

Historically, travel and exploration have been a male pursuit. However, since times immemorial, women also travelled, as a family or group member or a spouse. Women travels seem to have increased with the discovery, conquest, exploration and colonisation of other continents when women accompanied their menfolk. Records show that, for centuries, women also travelled solo [1, 2] for pleasure, studies and explorations – with enormous luggage and servants – to exciting destinations, many of which are inaccessible to the modern traveller. It is not known what advice was given to historical female travellers, but in 1889 a book with hints for the lady traveller catered for the growing number of women on the move [3]. Today, the ‘Recommendations for the Practice of Travel Medicine’ issued by the Faculty of Travel Medicine, Royal College of Physicians and Surgeons Glasgow (FTM RCPSG), Section 16 ‘Travel health issues for women’ [4], list a number of standards.

This paper considers women’s travel from three different but interconnected aspects. First, it discusses medical and health issues relating to those standards and offers corresponding practical suggestions. It then moves on to the increasingly important topic of safety and security including recent developments in cybercrime. Finally, no comprehensive contemporary exploration of travel aspects is complete without the application of mindful and responsible travel behaviour, here specifically of women, to minimise harm to fellow humans and the natural environment.

The following discussion applies to women travelling solo, in pairs or groups, with men, with a life partner, as an accompanying spouse/mother of a family, or visiting friends and family. Included are women who travel for work and those whose work is travel, e.g. flight attendants, as well as women from different cultural backgrounds. It will be self-evident which aspects apply to which type of traveller, situation or destination. Much may be of no relevance for the seasoned traveller, or the woman travelling to a modern city staying in a quality hotel for shopping and visiting art galleries or the theatre, or spending a few days on a luxury cruiser. Many of the discussed issues, however, will be of great importance to novice travellers and those who leave for trips to less developed, rural, remote or wilderness regions, including unpredictable transport options, plan to volunteer short or long term in rudimentary circumstances, or rely on homestays with poorer local residents.

## Method

The literature was searched using PubMed, Scopus, Web of Science, ScienceDirect, ProQuest, Google Scholar as well as grey literature and relevant websites with search terms:” female travel*”, “women travel*”, ‘pregnancy AND travel’, ‘contraception AND travel’, ‘menstruation’, ‘menstruation AND travel’, “menstruation suppression”, “female hygiene”, “urinary tract infect*”, ‘menopause AND travel’, “female sex tour*”, “risk perception”, ‘risk perception AND travel’, “solo travel*”, “travel safety”, “travel security”, ‘travel AND tattoo’, “pubic hair removal”, ‘cybercrime AND romance’, “human trafficking”, “touris* death”. Reference lists of obtained papers yielded further sources.

## Health and hygiene

General travel health advice is not discussed here apart from the comment that nuances in female biology may need to be considered more in travel medicine research as in medicine generally.

### Women specific travel medical advice

Women specific considerations usually cover aspects of the female lifecycle. A summarised overview precedes the larger part of this section, the practical issues female travellers face.

#### Contraception

Women using contraception should consider re-evaluating their method for an upcoming trip, especially if it takes them across several time zones or to unfamiliar environments, e.g. high altitude [5] and different activities. Contraception is rarely mentioned in consults even though a change to another of the many available methods may be appropriate. The combined oral contraceptive pill requires disciplined usage which can be under-mined by travel-related disruptions, or vomiting and diarrhoea. In addition, the venous thromboembolism risk increases [6]. Calculating late or missed dosages can be complicated when in different time zones. To avoid errors, some women take a second watch and adhere to their home schedule. Long acting reversible contraceptives may be appropriate for some trips. However, contraceptives do not protect from STIs, and condom use has to be discussed as well as emergency contraception.

The contraceptive pill is often suggested to suppress menstruation for convenience or for special occasions, such as honeymoons. However, there is still the possibility of ‘spotting’ (breakthrough bleeding), causing bother. Menstrual suppression has been studied in female hajj pilgrims for whom menstruation would make the performance of Umrah rituals impossible and so the entire journey prone to stress, anxiety and disappointment [7]. For obvious reasons, much research has been conducted on menstrual suppression in defence forces. Although some focused on operational benefits, i.e. the burden of menstruation on work performance, readiness and ability to deploy [8–10]. Others studied practical issues, especially hygiene challenges, in difficult environments of heat, dirt and rudimentary facilities [11] which mirror precisely the challenges travellers face in unforgiving circumstances and will be discussed later. The medicalisation of suppression is not welcome by all women [12, 13], a reluctance also reported by female soldiers. Hence, a tool was developed to measure attitudes towards menstruation and menstrual suppression [14, 15] within a theoretical model that places the woman in a microsystem of roles, activities and physical features (on deployment) [16]. This model applies easily to travel in rough and remote conditions.

#### Pregnancy

Relatively few papers are available on comprehensive advice for pregnant travellers, especially those to remote or wilderness areas [17–21]. Others focus on vaccine safety [22] or yellow fever and malaria prophylaxis [23]; the complex topic of the latter has been highlighted in a recent editorial [24]. Further discussions deal with air travel during pregnancy [25], venous thromboembolism [26], schistosomiasis in pregnant travellers [27], anti-helminthic treatments [28], or the potential exposure to rubella during a flight [29]. More recently, concerns about the Zika virus added another dimension [30, 31]. Cooperation with the pregnant traveller’s obstetrician is customary, also in relation to (un) planned deliveries abroad, breastfeeding and travel medications, as well as the reminder to contact the appropriate authorities in relation to a newborn’s nationality.

#### Menopause

Post-menopausal women travellers are not much considered in the literature. In a 1962 paper on aging and space, female space travellers were said to be lucky as they did not have to worry about menstruation, hygiene products, iron loss or radiation-induced genetic defects [32]. Back on earth, the recently introduced ‘genitourinary syndrome of menopause’ is relevant to older travellers as they need to ensure enough treatment provisions for the trip [33] as well as sufficient incontinence pads or underwear if required. Older (solo) travellers also need counsel on sexual behaviour, STIs and the use of condoms in preparation of unplanned and ‘out of character’ sexual activities with locals or other travellers [34].

#### Other issues

Surprisingly rare (or unreported) severe perineal injuries due to aeroplane vacuum toilets may increase in the future with toilets getting smaller, and not only overweight or obese passengers occlude the toilet creating suction [35, 36]. A device to urinate standing may be advised but a mandatory warning should avoid more injuries. With some airlines reducing the size of the actual toilet cabinet, remaining seated on the toilet while combing hair or fixing the make-up may be the only way to manage the diminishing space.

### Female traveller specific practical issues

A core concern for the travelling woman is the maintenance of her personal hygiene. This concern applies not so much to destinations in the industrialised world where facilities allow women to function like at home but to places that make a tried and tested routine difficult or impossible. However, a comfortable situation can change quickly, as has been seen in the sudden quarantines on luxury liners at the beginning of the COVID crisis, presumably with insufficient supplies.

#### Personal hygiene

Regular handwashing or sanitizing, and maintaining a clean body should be standard routine to protect oneself and others from infections. Often overlooked is cleanliness when travelling with contact lenses. Suggestions on hygienic contact lens use to avoid serious infections can be found elsewhere [37, 38]; a habit of using up old makeup and mascara while travelling may prove detrimental. Apart from general cleanliness, intimate hygiene is of particular interest to avoid genitourinary infections, expressly when facing unusual or difficult circumstances, more so in hot and humid climates. To maintain a healthy vulvar skin microenvironment, hypoallergenic, soap-free, pH friendly, non-irritant female intimate washes protect against dryness often caused by soap and balance the microflora [39]. These products are available as liquids or practical wipes. Travellers should refrain from trying out harsh local culturally based cleansing procedures. Cotton underwear is often advocated to stay fresh and dry. When sweat runs down the body and pools in underwear, cotton does absorb the moisture, but then retains it and maintains a wet environment – the exact opposite of what is required. The same happens in cold, wet situations where cotton underwear and thick jeans stay wet and cold for prolonged times, possibly contributing to the development of cystitis. Moisture wicking material may be a better choice. Superfine merino underwear with panty liners have been worn with great success (observed in trekkers, IB).

Panty liners are popular to ‘stay fresh’ and keep underwear clean. However, they can change the microclimate and promote bacterial and fungal growth. Conventional liners with plastic back sheets increase the skin temperature, moisture and skin pH significantly, and ‘breathable’ (= vapour-permeable) or acidified products are preferable [40–42]. Sharp edges on liners may cause microabrasions and increase the risk of infection. Perfume and the disinfectant Cu (II)-acetyl acetonate were linked to contact urticaria and dermatitis [43].

Urogenital (e.g. urinary tract, vulvar, internal genital) myiasis has been observed due to rudimentary sanitation and poor genital hygiene [44]. Female flies are attracted by malodorous discharge, e.g. urine or menstrual blood. The female *Cordylobia anthropophaga* (African tumbu fly) lays eggs on clothing hung to dry, *Wohlfahrtia* spp. on clothing on the ground [45, 46]. Long-term travellers and volunteers in endemic areas should be advised to iron towels and clothing, including underwear, on both sides. Less dramatic are possible candidiasis infections, especially in women on contraceptives or antibiotics, for which appropriate topical treatment should be packed.

#### Bodily functions

Fear of embarrassment or compromised modesty, depending on the traveller’s cultural background, as well as an actual or imagined lack of privacy or questionable cleanliness may make using toilets very stressful. Toilets with missing or not closing/locking doors, holes in cubicle walls, very small cubicles, wet sticky floors, long-accumulated dirt, lacking hooks for bags or clothing, missing lights, and so on – none of this an issue for the urinating man – need to be managed. Squatting toilets are hard on hips and knees, especially for older women lacking muscle strength or with arthritis, or for those keeping their backpacks strapped on for lack of (dry) space, similar to female soldiers in full combat gear [47]. A small headlamp for dark toilets or for outdoors at night is useful and preferable to a torch.

A number of gadgets allow women to urinate while standing to avoid touching unsavoury toilets, or for lack of protective undergrowth outdoors. Standing upright with the men in front of perplexed locals requires considerable self-esteem, but the gadgets are very useful in dirty toilets, also on local trains, to avoid contacting anything and to keep skirts or pants off a wet floor. Any devices should be practised beforehand. Lacking men’s option to urinate against a tree or a wall while clutching their luggage, solo travellers face the additional problem of having to leave bags unattended to use a toilet in an airport or bus stop. In extreme circumstances, for example, a long bus trip with no known stop useable for women, or for women who need to urinate often, wearing incontinence underwear as a temporary portable toilet can be useful. Urinating (and defecating) in challenging situations such as climbing expeditions [48] or in cold, wet, muddy, dark, overt circumstances should be planned, mentally prepared for (and practised) in advance. Best is to accept the challenge and ask others – who are in the same situation – to look away. That toilets play an important part in travel show volumes such as ‘How to shit around the world’ [49] with plenty of anecdotes, hints and tips on the ever popular topic of elimination.

In order to minimise the need for toilets, many travellers, as do military women [50], try to limit the intake of fluids and also ‘hold on’ until a more acceptable opportunity arises. Though understandable, such ‘prevention’, possibly combined with compromised hygiene, can lead to urinary tract infections (UTIs). There is still a lack of research on fever in the returned female traveller. A small study reports upper UTIs as the leading cause of fever among women returning from the tropics, possibly due to hot climate, long journeys, dehydration and poor hygienic conditions [51]. Urinating ‘just in case’ whenever possible is preferable to ‘holding on’ [50]. The use of self-diagnosis kits for genitourinary infections in military women has been tested [52] which might be useful also for long-term travellers, as could be the currently in development smart-phone based test to identify UTIs [53].

#### Menstruation

Menstruation is not a medical condition but can turn into a major nuisance when travelling. Travel can make periods lighter or heavier, longer or shorter, more unpredictable or disappear altogether due to stress, disturbed circadian rhythm, e.g. in female flight attendants [54, 55], or changes in climate and activities, e.g. in backpackers [56] or volunteers [57]. Women’s attitude to menstruation may determine their coping abilities. For example, negative menarcheal experiences may be linked to negative menstrual attitude later in life [58]. No research on menstruating travellers appears available to-date but tools such as the long-used Menstrual Attitude Questionnaire (MAC) [59] or the Military Women’s Attitude Towards Menstruation and Menstrual Suppression scale (MWATMS) [14] would be useful as travellers with more negative attitudes may support menstrual suppression for a trip. Menstrual suppression is considered frequently by travellers; however, with the practical inconvenience already discussed, it may be hardly worth the bother as sanitary products still have to be carried just in case. The relaxed approach of not using any sanitary products, as documented during a solo-crossing of Australia [60], is hardly applicable to regular travellers.

Four different sanitary products assist with menstrual hygiene. In contrast to home, many challenges can influence a normally straightforward procedure. Panty liners are often used before, after or during lighter periods. Disposable sanitary pads come in various shapes, sizes and absorption capacities to collect discharge. Sporadic research examined the presence of dioxin for bleaching fluff pulp in pads [61], the unpleasantness of pad surfaces, e.g. the wicking properties of mesh vs non-woven, and the reflux of discharge to the surface [62], an issue of interest to travellers, or dermatitis caused by pads [63, 64]. Tampons with or without applicator have been popular for a long time. A study on dioxin content was reassuring [65]. The serious toxic shock syndrome is now very rare [66] but care should still be taken to watch for symptoms, especially when far away from medical care. Menstrual cups have slowly gained popularity as a cost-effective reusable alternative which needs emptying, rinsing and re-inserting every 4–8 h. One can even get cups with an app to monitor proceedings in the device [67]. The benefit of disposable cups [68] is less evident. Menstrual cup insertion needs to be practised for a few cycles before travelling. A systematic review and meta-analysis reported leakage similar or less compared to other methods but also some negative outcomes [69]. An incorrectly placed cup may be irretrievable without assistance [70], a potential worry on the road.

Menstrual hygiene becomes a challenge when toilets are deemed unusable, the start of periods is unpredictable or the flow unexpectedly heavy. Potential ‘accidents’, i.e. bleeding through clothes is embarrassing, especially when remedial action is hours away. To prepare, many women use the largest sanitary pad combined with the largest tampon. However, the more blood accumulates, the more it provides a medium for bacteria to grow and produce odour over time [50]. The same occurs when a tampon is forgotten, and a second one is inserted by mistake (or to ‘double-up’ to avoid an accident) and an alarming foul odour alerts to the problem. The feat of a soldier changing a tampon in a driving tank [47] is admirable but not the action of choice for a traveller, e.g. in an overnight bus. Pads stick poorly in heat and sweat and so may cause leakages. Changing menstrual cups in grubby toilets may lead to spillage over hands, clothes or the floor; a small water bottle needs to be carried to rinse the cup. The lack of hand-washing facilities when changing sanitary products, especially tampons, has been identified [47, 71]. Wipes may be a quick solution but running water and soap deal best with blood-stained sticky hands. Many women like to keep their periods secret and are embarrassed by dealing with changing and disposing of products, or drying blood-stained underwear in hotel bathrooms or on clothes lines between tents. Recent reports on the visibility of sanitary products on Transport Safety Administration (TSA) full body scanners at airports and subsequent upsetting treatment of the ‘suspects’ [72, 73] needs more clarification than a statement by the TSA [72]. The official TSA website (www.tsa.gov) does not seem to offer this. On a practical note, ideally, keeping all supplies in a bum bag (preferable to a transparent Ziploc bag), such as sanitary products, toilet paper, hand wipes, female wipes, soap leaves or mini bottle of liquid soap, and a few sanitary disposal bags to separate used and unused items, allows easy access. On the road, and to be prepared for an unintentional separation from the luggage, e.g. an evacuation from an overnight bus due to a landslide, it is best to keep supplies on the body. Despite a study on polar bears’ interest in menstrual odours [74], there is no evidence that sharks and other predatory animals are keen on menstrual blood.

#### Sexual behaviour

Many women have sex during travels with fellow travellers or local partners. The literature seems only mildly interested in sex among travellers; on the other hand, much has been written about female travellers’ sex with local partners, a phenomenon long in existence but only more recently acknowledged [2]. Being away from home gives some women an unexpected feeling of freedom which allows them ‘the unthinkable’ adventure with a local partner, leading perhaps to an instant romantic episode [75]. Others travel for the specific purpose of having sex with locals, even considering a ‘brown baby’ as a souvenir [2]. In both situations, alcohol and drugs may lead to imprudent decision-making regarding safe sex but also compromise a traveller’s safety and security. Travel health advice includes discussions on the importance of condom use and strategies after unsafe sex.

#### Psychological travel health

Mental health issues in travel are woefully under-researched. Psychological wellbeing applies to any aspect of travel; only few have been discussed in the literature, none is examined in-depth, with gender a decisive but overlooked factor. More work has been conducted on long-term travellers, e.g. expatriates or volunteers, mainly on men. Women often rate a mention as accompanying spouse or mother whose feelings of boredom, isolation, alienation for not fitting into the host country’s concept of the ideal woman, are acknowledged anecdotally. Having to tolerate a husband’s adjustment to local requirements of heavy drinking after work and sexual indiscretions [76], or being unable to work despite being highly qualified, may lead to depression or anxiety, blaming country and people for all ills. Not coping well with (sometimes unwanted) changes of relocation, marital disharmony or reproductive concerns may make an overseas deployment torture for all involved. Younger volunteers may lose their initial sense for adventure when homesickness sets in, the famous culture shock, or difficulties with the practicalities of life, such as hygiene, food, lack of privacy during homestays including relaxed local attitudes towards ectoparasites [77]. The need for pre-travel mental assessment and post-travel debriefing has been highlighted before [78, 79] but not all problems can be foreseen. Difficulties in readjusting to life back home, a ‘reverse’ culture shock, and the realisation that people at home are not that interested in one’s overseas experiences, nor are potential employers, are distressing for many returning women.

Leisure travellers may also experience distress and anxiety. Mental health issues in air passengers and crew [80] and especially a fear of flying [81, 82] have been discussed, the latter suggesting a higher prevalence in women, although there seems to be no more recent evidence. On the ground, distressing worries about privacy and hygiene but also culture shock, homesickness, loneliness and unfamiliar or frightening ways of going about daily lives may trouble some women while others thrive on these challenges. For obese travellers, shame, humiliation and embarrassment for the inability to do simple travel-related activities, especially evident in air, train or bus travels, or for not displaying a pleasing body shape can ruin an otherwise enjoyable trip [83]. For some travellers with psychiatric problems, repatriation may be necessary [84]. On the other hand, travel can do much for mental wellbeing, either by enjoying sights and sounds of a destination, a particular mode of transportation, or by feeling a sense of achievement after completing a particular feat or to balance out a personally difficult time. Women travelling for a sport event displayed improved wellbeing [85]. Links between risk-taking and personality traits have been suggested [86] including their application to travel risk perception [87]. Apart from cultural differences [88], gender influenced travel risk perceptions and subsequent international travel intentions [89]. Unsurprisingly, for many women, the need to be particularly weary about their personal safety, a topic discussed next, may have them travelling constantly on edge to the point that enjoyment is replaced by fearing the worst.

Traditionally, and originating in the West, travel medicine has catered for the Western tourist. This focus has expanded recently with the inclusion of migrant health but it seems not yet to consider VFR travellers from developing countries visiting emigrant family members and friends in Western countries. These women may differ in skin colour, facial features and dress, and may be mistaken for immigrants and asylum seekers. Veiled and non-veiled tourists in industrialised countries suffer a reversed (not reverse) culture shock at destination [90]. Immigrant and religious stigmatisation [91] spoils their tourist identity [92], leaving the women with feelings of injustice, shame, insecurity and the desire to go home.

## Safety and security

Centuries ago, immediate danger threatened from unsafe roads, inclement nature, untrustworthy modes of transportation, unsavoury fellow-travellers, wild animals and hostile ‘natives’. Many women will have met with misfortune but many others have returned home to tell the tale. With increasing female education and literacy over the last centuries, women’s travel literature, especially of the 19th and the beginning of the 20th centuries, testifies to the often dangerous episodes and how the women overcame them. Today, there are contemporary versions, which equally need to be addressed. This section focuses on the FTM RCPSG standard ‘personal security’ [4] and presents aspects of women’s travel safety and security, suggesting ways to minimise potential risk. Safety generally means protection against unintended threat while security protects against deliberate harm. This paper does not distinguish, as carelessness in one can jeopardise the other, and many overlaps exist.

### Protection from unintentional harm

Safeguarding one’s health goes beyond clean food and water. Enthusiastic travellers often return home sporting a temporary souvenir, such as braided or beaded hair or red henna decorated hands. A rare case of contact dermatitis has been reported after a temporary black henna tattoo due to added para-phenylenediamine [93]. Perhaps in an altered state of mind, obtaining a permanent tattoo or a piercing in a local salon with unknown safety standards seems a good idea but exposes the traveller to a range of surface or systemic infections including sepsis or HIV. The current trend of pubic hair removal at home, or as an exciting novelty on destination, may expose women not only to infections due to microabrasions, microtears or burns during the procedure – and delayed healing in hot and humid climates – but also to STIs [94–97]. Pubic lice infestations, however, may decrease [98]. Other self-inflicted risks occur when trying out substances such as mescalin or ayahuasca [99] or suffering methanol poisoning after sampling cheap local alcohol [100].

### Protection from intentional harm

The main concern, however, is being subjected to harm by others. In most places, travellers are easy targets for their skin colour, casual clothes, and tourist gadgets. They also have to deal with the ‘stigma by association’ [91] which links them to the behaviour of female tourists who visited before them. Solo travellers may feel particularly vulnerable [101]. Women have travelled solo for a long time – and written about it [102]. The social construct of womanhood with a set of standards of femininity in the late 18th and the 19th centuries [103] did not include women taking off on their own, imitating men, and getting in harm’s way. Only more recently was the transgression into the male domain of (dangerous) adventure tourism challenged by women [104]. Today, travelling solo is still difficult for many women. In a Norwegian study, the majority of female university students found solo travelling frightening [105]. Australian women revealed four categories of constraints to solo travel – pre-travel and on location: socio-cultural, personal, practical and spatial [1]. Their fears were: others’ perceptions of them, being prone to vulnerability in unknown spaces, having a sense of restricted access and temporal mobility, as well as the feeling of conspicuousness, i.e. proneness to the male gaze [106]. Very recent papers focus on the Asian solo traveller, an increasing market segment, reporting fear and vulnerability [107], gendered risk (from wolf whistling, stalking, groping to rape), racialised risk (outside Asia), and being mistaken in Asia for local sex-workers, as Asian women are not supposed to travel alone [108]. India was singled out as a place to avoid [109, 110], especially after much publicised rapes [111]. This fear is not unfounded with non-partner violence common around the world [112] and tourist hotspots providing unwelcome happenstances [113]. Even in-flight sexual assaults have been reported [114]. Danger to life and limb is real as sporadic media reports of missing or murdered travellers highlight. Travelling solo makes some women more vulnerable in certain circumstances but they are also found to be often much more careful by avoiding dangerous situations. Women travelling with one or more females for the purpose of ‘having fun’ and ‘having each other’s back’ may engage in riskier behaviour due to a false sense of protection from the others.

Travellers’ risk perception usually sees the traveller as the victim of adversity [115]. Scales have been used to predict risk behaviour [116]. However, much happens during travel ‘out of character’. Conscious risk-taking is not well researched, for example, why some women accept or actively seek high risk during travel. Being in a liminoid time-space is an explanation for bad behaviour, not an excuse. Physical and sexual attacks are the biggest danger for any women, solo or otherwise. Appropriate behaviour can minimise the risks. Being realistic and stating the obvious is not always welcome in the current climate. The popular statement ‘it is never a woman’s fault’ is unhelpful as, at home and away, it absolves women from the responsibility of making prudent choices, including maintaining mental alertness, undulled by substances.

Women can take many precautions to travel safely. In preparation of the trip it is important to understand the destination (culture, standards) as well as oneself. Introspection should uncover any traits that might lead to unwise decisions. The trip’s purpose should align with the preparation, and information always left with friends and family at home. ‘What if...?’-scenarios need to be planned, e.g. adverse events, sudden dangerous situations, and so on. Several operators offer young single women the opportunity to accompany rich men on luxury travels for free. These arrangements are not free. Anxious or inexperienced travellers may feel safer with female-only operators.

General statements of ‘taking care’ or ‘being careful’ are not helpful; more specific hints usually are, especially for women who normally live in safe and protected circumstances. The following few key areas are supported by selected suggestions in Tables 1, 2, 3 and 4. Not all these hints apply everywhere and at all times; not every woman will need all or any. Women cautious at home will implement them by default; novices should take note. Planning transport and accommodation at least for the first days safeguard the start in an unfamiliar environment. Precautions for accommodation depend on the type of dwelling and the area in town as well as physical factors of building and surroundings (Table 1).

**Table 1:** Transport and Accommodation

**Transport**	
Avoid night travel; avoid arriving at night	
Arrange airport transfer to hotel in advance	
Use licensed taxis (know what they look like)	
Sit behind the driver	
Make ‘phone call’ in taxi to say you arrive shortly	
Pay taxi while still in the car	
On public transport, sit near women, use ‘pink tuk tuk’, lady taxis	
Self-drivers: Park so that you don’t have to back out	
**Accommodation**	
*Booking*	
Pay more for safety; avoid ground floors; choose ‘women floors’; avoid dorm beds or camping solo; swags restrict movement in case of an attack	
Book without title Miss, Ms., Mrs	
Avoid couch surfing with a male host	
*Arriving*	
Check in without title	
Check phone is working, lockable door, chain at door, peephole	
*Staying*	
Use a rubber doorstop if necessary	
Keys: best unmarked swipe keys; cover key room number in restaurant; avoid saying room number loud at reception/restaurant	
Don’t get into the elevator with a stranger	
*Leaving*	
Write down hotel name and address in local language	
Leave note in hotel where you go that day (but ensure that no unauthorised person hears in)	
Don’t discuss in public where you are staying

**Table 2:** Appearance and Behaviour

**Appearance**	
Dress modestly, not provocatively	
Wear pants rather than skirts (or pants/leggings under skirts)	
Observe the local dress code → check what local women cover	
Wear headscarf where appropriate	
Consider wearing a wedding ring and carrying a photo of a fake husband	
**Behaviour**	
*Sensible Behaviour*	
Stay alert	
Stay in well-lit, populated areas	
Keep low profile; try to blend in; walk confidently, with a purpose	
Enter a shop or business if there is a threat	
Don’t try-on clothes in bazaars, suqs, markets	
Don’t wear noise-cancelling earbuds or music ear buds	
Conceal your map/guidebook in the street	
Control your own behaviour	
*Communication*	
Make sure your mobile phone is always freshly charged	
Put local police on speed dial	
Stay connected as a precaution	
*Other*	
Learn a few basic phrases	
Always know your escape route	
Check sunrise and sunset times	
Be wary of travel companions who behave imprudently	
There is safety through protective guest family or person of authority

**Table 3:** Interpersonal Safety

**Plan ahead**	
Find out gender issues and attitudes towards women at the destination	
What are acceptable manners? Is it appropriate to approach men for questions? Does looking into men’s eyes mean an invitation? Shake hands?	
Do you know yourself enough? What are your plans in case …?	
Could your travel plans expose you to risks?	
- Shamanic experiences; sexual assault linked to ceremonies	
- Sex/romance tourism: more problematic where women tourists are new – local men don’t know how Western women function	
- Legal situation regarding adultery, pre-marital sex	
**Encounters**	
Don’t invite visitors to your room; don’t accompany men to their room; meet in foyer or in public places	
Avoid using local dating apps	
Don’t accept rides with strangers	
Understand local perception of Western women	
Limit alcohol, avoid any drugs; be aware of drink spiking	
Be alert at ‘full-moon parties’	
Be alert in crowds	
Don’t eat/drink somebody’s food if they don’t eat the same	
Don’t discuss travel plans with strangers	
Be aware of people posing as police or tour guide	
Control your own behaviour	
Know procedure in case of rape (police reports, forensic examination etc)	
**Protection**	
Wear a whistle, ‘rape alarm’	
Carry personal protection devices if legal at destination

**Table 4:** Safety of Property

**Luggage**	
Ensure luggage not too heavy to slow you down	
Use luggage in bold colours, personalise heavily or ‘uglify’ to make it easily traceable and unsellable	
Use covered luggage tags; use office rather home address	
Be alert in train sleepers, on bus	
Theft-proof bags (steel wire in handle/strap)	
Wear shoulder bags across body	
Keep handbag away from street side	
**Documents**	
Plan how to carry documents and copies of your passport, visa, prescriptions, ticket	
Don’t make copies of your credit card; memorise details	
Keep your identity safe	
Avoid cargo vests for carrying money and documents; they can be ripped off easily	
**Money**	
Understand money notes, coins and exchange rate beforehand	
Use ATMs during the day; bring enough cash for the first days	
Be careful with anything where you need to get your wallet out	
Use dummy wallet with little cash	
Use karabiner clip wallet; sew safe pocket under bra or inside pockets in pants	
Use travel ‘safes’ (money belt, money pouch, leg pouch etc.) but ensure none shows through clothing	
**Stay alert**	
No distraction	
Secure luggage, laptop, tablets, personal information	
Flashing a laptop or tablet in public may invite robbers	
Don’t resist when robbed; hand over valuables	
**Watch out**	
Watch out for scams targeting the female traveller (e.g. by women with babies or group of children)	
Learn about current pickpocket methods and scams	

Appearance and behaviour are important aspects of travel, not only to show respect to the host country but as a precaution against harm. Many women claim it their right to wear whatever they like where they like and behave in whatever way they wish since they are on holidays and paid for their enjoyment. This opinion has nothing to do with personal freedom and contradicts common-sense. What body parts local women cover is a good guideline as local mores may require covered shoulders, arms, or knees. Arguing with a guide about different perceptions of when a knee is covered is embarrassing and displays a lack of understanding and respect. Table 2 lists a few suggestions.

Interpersonal safety aspects concern interactions with others (known and unknown) to minimise harm to life and limb. Prudent and cautious interactions are asked for, not paranoid reactions. Misfortune often strikes with the involvement of alcohol or drugs and the abandonment of natural instincts. In addition, inebriated female tourists losing self-control do not evoke a high opinion in locals. Women whose behaviour is interpreted as invitation or provocation, or which makes them the object of ridicule, are not safe. Table 3 presents a list of suggestions out of a great many more. Female travellers should also have a plan of what to do if they are assaulted or how to get out of an increasingly dangerous situation. Members of local authorities are not necessarily trustworthy.

The safety of one’s possessions is of importance as theft or damage not only ruin a holiday but can eventually jeopardise personal security. Female travellers are often scammed by women using ‘sisterhood’ as a cloak. Table 4 presents a list of hints but women should be aware that criminals are usually one step ahead, know all precautions and change their mode of operation accordingly. Fake soiled lingerie, prominently displayed on top of backpacks or suitcases, can keep some long fingers away.

Women are now the main victims of online romance scams [117]. This crime progresses through stages [118], involves transfers of money and often travel to the country of the cyber-partner. Women then find that not only does the man not resemble that expected partner but themselves involved in criminal activities, such as money laundering or drug muling. They may be kidnapped [119], or become a victim of human trafficking, recruited by their trusted ‘boyfriend’ [120, 121], an occurrence even in Western countries [120, 122].

Finally, harm can be the purpose of travel. Arranged marriages (especially under-aged girls) are prone to inflict physical and psychological harm. Girls travelling for a ‘special celebration’ should alert a travel health consultant. Female genital mutilation (FGM) has received much greater attention in recent years, even though how to deal with the legal requirements and overcome barriers to discuss are less clear [123–125]. Some guidelines are available [126] as well as a free eModule from the FTM RCPSG [127]. FGM may not yet be part of general practitioners’ perception of risk for VFR Travelers [128].

## Sustainable development and responsible travel

Women’s unique travel-related needs also highlight women’s specific role, obligation and opportunity within the context of responsible travel beyond the general guidelines as they apply to all travellers. Historically, travel was more about consumption rather than consideration (in 1889, women were encouraged to simply throw soiled linen overboard [3]). Never before has the demand for considering the impact of one’s travels, mindful and responsible behaviour, and sustainable actions been greater than today. Genuine conviction or compliance with political correctness, travel operators are expected to demonstrate their reduction in harm caused by travel and travellers. The International Society of Travel Medicine’s (ISTM) recent interest in the UN-Sustainable Development Goals (SDGs) [129] may lead to some alignment between the sustainability paradigm and travel medicine. While the SDGs have been written for governments and global organisations, travel clinics, travel health practitioners and travellers can play their part in contributing mindfully to the overall goals. The aim is to avoid a burden on destinations, especially fragile and vulnerable environments, and a negative impact on local lives and livelihoods.

The notion of a ‘shared prosperity in a sustainable world’ [130] is alluring, highlighting the two main foci: poverty and environment. Only in exceptional cases does tourism reduce poverty and so improve the health of the poor as the *status quo* is in the interest of many powerful stakeholders [131]. Climate change and the environment are on the forefront currently, yet, much of ecotourism is not at all ‘green’. Some years ago, travel medicine touched on the concept of OneHealth [132], the health-interrelationship between people, animals and environment. The interest in people’s health is obvious, in environmentally friendly travel mild, but the role of travel medicine in animal welfare is minimal [133].

Responsible travel is mindful travel turning exploitation into consideration. Destinations, especially poor countries, have no obligation to provide a playground *and* clean up after us. Economic, environmental, socio-cultural and political impacts of tourism have been discussed from the 1960s onwards. These recognised impacts’ influences on local health and wellbeing as well as tourism’s direct health impacts, such as transmission of infections, accidents, and occupational health and safety, were only considered from the mid-1990s [134]. Within the ISTM in the 1990s, the then ‘Host Countries Committee’ (now ‘Responsible Travel Group’) began a fringe-existence as a curiosity; the need to care for the hosts is now accepted. This section suggests to female travellers actions that can contribute to responsible travel, careful consumption and waste disposal, and appropriate conduct, by first addressing the established overall tourism impacts and then focussing on women-specific practical aspects.

### Mitigating negative economic impacts

To assist the local economy, and acknowledging that families enjoy a better standard of living when women are in employ, travellers should spend locally on accommodation, transport, food, craft (not foreign copies), and avoid aggressive haggling. Female tourists seem to buy more souvenirs than men [135]. Women can enter local homes more easily and may be able to buy directly from craftswomen. In some countries women face barriers to participating in the tourism industry [136]; elsewhere, women can benefit directly from tourism, often as small business owners. Supporting local women helps overcoming gendered disparity and cultivate women-to-women interactions, for example, in the case of female guides in Nepal [137].

### Mitigating negative environmental impacts

Environmental mindfulness includes supporting local environmentally beneficial efforts, such as low carbon transport and the use of filtered water over plastic bottles. Beauty regimes can be upheld with less water, where scarce, and ecofriendly cosmetic products. Specific waste disposal will be discussed in the relevant sections later in this paper. Gifts should be more useful than burdensome. Donations’ end-of-life disposal needs consideration: soaps and shampoo as bars in paper or cardboard instead of plastic wrappers, no plastic toothbrushes, no small hotel bottles, traditional good quality pencils instead of plastic casings, and so on. In short, polluting gifts should not turn locals into polluters.

### Mitigating negative socio-cultural impacts

Female travellers have a unique opportunity to bridge cultural distance and maximise socio-cultural understanding by making friends with local women. Visiting homes (invited) and exploring local life through local women’s eyes counteract perceived differences [138]. Respectful behaviour excludes aggressive promotion of Western values or patronising and ridiculing disapproval of challenging customs, such as geophagy [139] or decorative scarring. It excludes crossing the line by requesting unacceptable favours, such as breaking taboos, which create (intergenerational) family or community tensions as does singling out individuals for gifts or donations. It is better to donate to schools or health centres, for the benefit of all. Local children in ‘orphanages’ or local sexual partners are not playthings [140]. Purchasing souvenirs that demean local women is not responsible; female travellers have no automatic right to photograph local women without their consent. Many famous resorts around the world, e.g. Boracay in the Philippines, have been built on ancestral land after removing the indigenous population with a particular devastating impact on women and children. Researching the history of one’s destination and possible rectification strategies should guide a responsible choice of one’s holidays.

### Mitigating negative health impacts

Following the above suggestions, female travellers safeguard indirectly local people’s mental and physical wellbeing. However, their behaviour can also minimise direct risks to local health. Personal cleanliness and social etiquette, mindful disposal of waste and sanitary products and safe sex protect local hosts, whose health may be compromised due to malnutrition, parasites or infections, or limited finances to seek health care. Driving with care and other considerate behaviour avoids harming locals. Workplace health and safety regulations are often non-existent. Using ‘delicate femaleness’ as leverage is not responsible behaviour. Travellers should not ask for excessive services, e.g. carrying heavy backpacks [141], dangerous or illegal activities or unreasonable favours, but rather inquire about and check what operators do to protect the local workforce. It helps being aware that one’s enjoyment is often only possible by exploiting a local workforce or local people.

### Women-specific practical aspects of responsible travel

Guidelines for responsible travel are ubiquitous but rarely go beyond general statements. Detail of how to implement those guidelines is normally missing. This section provides such detail linked to some core concerns of female travellers. The main focus is on waste disposal and on appropriate behaviour.

#### Personal hygiene

The responsible traveller washes her hands, coughs into her elbow, sneezes into tissues, disposes mindfully of contaminated products and so assists in curbing the spread of respiratory and gastrointestinal infections to local residents. Half-empty containers of shampoo, washing liquid and so on may be happily accepted or empty containers repurposed by local women; otherwise, bottles should enter a recycling stream or be taken back to the best facility for disposal. Wipes may be able to be burned but must not be thrown into toilets or left in the outdoors (also not buried). Regular panty liners are hard to recycle and should not be discarded but carried back. Small zip lock bags can cater for such waste.

#### Bodily functions

In many regions, local plumbing does not accommodate toilet paper which should be deposited in the provided receptacles. In the outdoors, toilet paper should be burned or carried out, never left on the ground or buried. Especially for defecation in the outdoors, there are specific rules about where and how deep a hole needs to be dug. Covering faeces with leaves or rocks attracts animals and is unsanitary, especially in the case of infectious diarrhoea. Disposable urine gel receptacles are useful in some situations but not in areas without proper disposal management. Incontinence pads and underwear should equally be carried out and dealt with responsibly.

#### Menstruation

An innate urge to disassociate oneself from waste by stepping away quickly may make otherwise sensible women do bizarre things. Sanitary products must not be disposed of in toilets and should not add to the burden of the local waste disposal system. If fluff can be burned locally, the plastic casing needs to be removed and disposed of responsibly (by carrying out). Sanitary products should not be buried in the outdoors as animals can dig them up, tampons not flung into the bushes. The use of reusable pads and their washing and drying may be something to get used to but avoids adding to the enormous landfill, i.e. up to 26 kg of pads over 10 years/woman [69]. Tampons without applicator are preferred or at least those with paper applicators. Plastic applicators, popular in the US, cannot be recycled and should be carried out, similar to applicators for vaginal moisturisers or topical treatments. The recent keen market for used sanitary products to produce hallucinogenic drinks [142, 143] is of limited use to the travelling women.

The importance of menstrual hygiene products for girls and women in developing nations has attracted recent interest [144, 145]. Sanitary products are not just pieces of pulp absorbing blood. They open the door to life-changing education for girls around the world who are unable to attend school because of a culturally imposed local stigma. Managing menstrual hygiene is ‘an issue of social justice within the context of public health’ ([145],p. 1302). Many campaigns distribute disposable products in low-income countries (see ‘28 May Menstrual Hygiene Day’ [146]), though their disposal is still problematic. Donating reusable pads to visited communities may be a better option [147]; local women’s views on washing and drying them where menstruation is a taboo and a matter of shame and discrimination, need to be ascertained first. As always, donating without considering the end-of-life cycle of the item and the burden of disposal, is not responsible.

#### Sexual behaviour

The responsible traveller not only protects herself from STIs but also her partners by using condoms. However, not only physical health is implicated. Local men available for tourist sex can be resented by their families and local society as they do not follow cultural script. Displays of affection in public initiated by travellers may upset community standards, and a destination can acquire an unwanted reputation. More details on local impacts of sex between female travellers and local men can be found elsewhere [2]. Contrary to common belief, not all men are romantic entrepreneurs. Some suffer mentally when their temporary girlfriends leave perhaps forever [140].

#### Psychological health

A mentally unwell traveller affects her fellow travellers as well as local hosts. The manifestations of the problem, e.g., verbal or physical aggression, mood swings or deep sadness, can cause anguish, offense, disappointment and misunderstandings. Unfriendliness, belittling of customs, rejecting well-meaning help, criticising local lifestyle or food may be perceived as personal or cultural rejection. The way a Western woman expresses her unhappiness may be puzzling or offensive to others who themselves are then affected.

#### Respect

The responsible traveller shows respect for the hosts in comportment and dress, especially in places of worship (churches, mosques, temples, sacred sites). Visits to Islamic countries challenge some Western women. While Muslims have a traditional obligation to offer hospitality to strangers, tourists need to abide by the rules respectfully. Islam encourages tourism but many countries do not offer clear dress codes for female tourists. While Iran and Saudi Arabia have guidelines [148], in Egypt, for example, female dress codes vary between progressive and conservative families, and resentment to scanty clothing extends to tourists, whereas in Malaysia, rules for Muslims are strict, for non-Muslims somewhat relaxed [149]. The mindful traveller also avoids eating her meals or drink in public during Ramadan. Fitting into local female norms [150] for a limited time allows a deeper insight into a destination as difference is one appeal of travel. Showing respect, female travellers will be respected back.

## Recommendations for education

Experienced women have learned over time and frequently share their advice on relevant travel websites. Oftentimes, common-sense just needs some additional hints and reminders. A standard travel consultation rarely affords the time for a comprehensive discussion of topics beyond the specifically medical issues. Detailed suggestions relating to the three main areas covered here should not end up as a politically correct exercise buried in academic papers, only accessible to a limited number of readers. International travel medicine organisations, such as the ISTM and the FTM RCPSG, and regional organisations should make travel health advice more accessible to the travelling public via women’s magazines, blogs, and women-specific travel sites such as Journeywoman.com. They could make available to clinics, travel health professionals and the public downloadable guidelines, which should be updated regularly. ISTM’s Responsible Travel bookmarks and flyers published in the early 2000s were a useful start but, time progressing, advice needs to be updated, amended, and new developments in types of travel, tourist activities and shifting interest incorporated. Assistance should also be provided to local accommodation and hospitality as well as local tour operators so that women can be given locally relevant advice.

The problem with ‘compliance’ with health advice is not new [151]. The responsibility of implementing behaviour change is placed with the individual after somebody else has instructed what needs to be done. Doubting the ability of the individual to control behaviour based on causal and moral responsibility, a recent discussion included the notion of prudence into health promotion, i.e. following advice because it is in accord with people’s own interest in their health and wellbeing [152]. This approach could be explored more in travel medicine.

## Recommendations for research

Women specific research in travel medicine is relatively scarce; women’s views on and experiences of many travel-related women-specific issues seem completely neglected. Much research in travel medicine and tourism focuses on travellers’ attitudes, yet fails to address the highly complex nature of that concept [153] which, again, should be explored gender-specifically because it impacts on health, safety and responsible behaviour.

This article contains many potential research topics. In relation to health and hygiene, of particular interest is an insight into travel-related barriers and challenges unique to women and the degree of success in coping with them. Topics include aspects of the female lifecycle, such as contraception, menstruation (suppression), pregnancy, menopause and sexual health. Further areas are practical issues relating to personal hygiene, bodily functions and potentially yet undiscovered topics. Mental health issues are largely ignored, as are challenges for the accompanying wife or caregiver of children and other dependent family members. An understanding of women’s specific expectations from travel consultations should prove useful. Some defence force research, while not necessarily transferable, may suggest further topics, also for comparison’s sake.

In terms of safety and security, not enough is known about the health outcome of women-specific body alterations made during travel (excluding medical tourism). (Solo) female travellers may report particular yet unknown travel health and safety concerns and a corresponding need for inclusion into travel health consultations. More insight is required into a women-specific association between risk perception and behaviour during travel and at destinations. While there is a large body of knowledge in tourism academia on local people’s perceptions of tourist behaviour, more insight is needed into locals’ perception of female travellers’ safety and security.

Topics within the context of responsible travel, from a female perspective, seem completely neglected. Even recent research on sustainable travel behaviour does not differentiate between genders, a difference one might assume when it comes to towel and linen reuse in hotels [154, 155]. This lack of evidence provides an empty canvas for numerous research topics arising from this paper. There is a lack of knowledge about women’s understanding of responsible travel behaviour and their possible need for more information. A more specific focus on locals’ perception of behaviour and conduct of female travellers, especially in traditional societies, present a further opportunity for collaborative investigations. Research on women-only tours and with solo travellers, in relation to responsible travel behaviour, but also safety and security, may yield unexpected insights.

Cooperation with tourism researchers should pave the way for more multidisciplinary research on topics of mutual interest [156]. Validated research methods from other disciplines can assist where topics are outside the applicability of traditional medical and positivistic protocols, for example, research on location with smartphones [157, 158].

## Conclusion

For most women, going on a trip involves a number of medical preparations as well as careful planning of practical aspects around hygiene, yet, there is scarce discussion in travel medicine about the travelling women’s needs and challenges outside traditional topics. This paper offered a long overdue presentation of three distinct but interconnected aspects of great importance to these travellers, including suggestions for education and much needed further research. First, it provided a much more detailed exploration of travel health and hygiene than usually given. However, for women, the preparation of a trip involves more than just strictly medical considerations. Safety and security are of concern when venturing outside the perceived protection of the familiar realm. The excitement of travel may lead to unwise decisions based on an altered perception of risk. Harm can be self-inflicted or inflicted by others. Not following prudent advice does not necessarily lead to misfortune but smart behaviour allows women to have a safe and enjoyable trip. A range of common, potentially unsafe situations include the more recent cybercrime. Finally, the concept of responsible travel is not new to travel medicine but its acknowledgement has rarely progressed beyond general statements and suggestions, even though questionable practices in a range of travel situations are well known. This paper presented responsible behaviour in a more detailed and practical sense, applied to women as a specific travel population. The individual female traveller cannot compensate for large-scale inaction but she has many opportunities to avoid aggravating local problems. ‘Doing the right thing’ may be difficult or impossible at times, but striving for it allows women to travel while safeguarding fellow humans and the natural environment.

## Data Availability

Not applicable.
